# SARS-CoV-2 Subgenomic RNA Kinetics in Longitudinal Clinical Samples

**DOI:** 10.1093/ofid/ofab310

**Published:** 2021-06-11

**Authors:** Renu Verma, Eugene Kim, Giovanny Joel Martínez-Colón, Prasanna Jagannathan, Arjun Rustagi, Julie Parsonnet, Hector Bonilla, Chaitan Khosla, Marisa Holubar, Aruna Subramanian, Upinder Singh, Yvonne Maldonado, Catherine A Blish, Jason R Andrews

**Affiliations:** 1 Division of Infectious Diseases and Geographic Medicine, Stanford University School of Medicine, Stanford, California, USA; 2 Department of Epidemiology and Population Health, Stanford University School of Medicine, Stanford, California, USA; 3 Departments of Chemistry and Chemical Engineering, Stanford University, Stanford, California, USA; 4 Department of Microbiology and Immunology, Stanford University School of Medicine, Stanford, California, USA; 5 Department of Pediatrics, Stanford University School of Medicine, Stanford, California, USA

**Keywords:** cohort, COVID-19, infectiousness, SARS-CoV-2, subgenomic RNA

## Abstract

**Background:**

Given the persistence of viral RNA in clinically recovered coronavirus disease 2019 (COVID-19) patients, subgenomic RNAs (sgRNAs) have been reported as potential molecular viability markers for severe acute respiratory syndrome coronavirus 2 (SARS-CoV-2). However, few data are available on their longitudinal kinetics, compared with genomic RNA (gRNA), in clinical samples.

**Methods:**

We analyzed 536 samples from 205 patients with COVID-19 from placebo-controlled, outpatient trials of peginterferon Lambda-1a (Lambda; n = 177) and favipiravir (n = 359). Nasal swabs were collected at 3 time points in the Lambda (days 1, 4, and 6) and favipiravir (days 1, 5, and 10) trials. N-gene gRNA and sgRNA were quantified by quantitative reverse transcription polymerase chain reaction. To investigate the decay kinetics in vitro, we measured gRNA and sgRNA in A549^ACE2+^ cells infected with SARS-CoV-2, following treatment with remdesivir or dimethylsulfoxide control.

**Results:**

At 6 days in the Lambda trial and 10 days in the favipiravir trial, sgRNA remained detectable in 51.6% (32/62) and 49.5% (51/106) of the samples, respectively. Cycle threshold (Ct) values for gRNA and sgRNA were highly linearly correlated (marginal *R*^2^ = 0.83), and the rate of increase did not differ significantly in the Lambda trial (1.36 cycles/d vs 1.36 cycles/d; *P* = .97) or the favipiravir trial (1.03 cycles/d vs 0.94 cycles/d; *P* = .26). From samples collected 15–21 days after symptom onset, sgRNA was detectable in 48.1% (40/83) of participants. In SARS-CoV-2-infected A549^ACE2+^ cells treated with remdesivir, the rate of Ct increase did not differ between gRNA and sgRNA.

**Conclusions:**

In clinical samples and in vitro, sgRNA was highly correlated with gRNA and did not demonstrate different decay patterns to support its application as a viability marker.

Understanding and quantifying the replicating or transcriptionally active virus among individuals with severe acute respiratory syndrome coronavirus 2 (SARS-CoV-2) could inform treatment decisions and response monitoring, as well as the need for isolation, contact tracing, and infection control measures. The duration of infectiousness as estimated from transmission studies appears much shorter than the duration of polymerase chain reaction (PCR) positivity in airway secretions [1, 2]. Studies comparing culture and reverse transcription PCR (RT-PCR) from the same samples have revealed that there is often substantial discrepancy between these measurements, with PCR remaining positive for days to weeks longer than culture [3–7]. While culture remains the reference standard for detection of infectious virus, it may lack sensitivity, and it requires biosafety level 3 facilities, precluding its use at scale as a clinical or public health tool [8]. To overcome this obstacle, there has been considerable interest in the development of molecular viability markers to sensitively detect and quantify transcriptionally active virus [3, 9, 10].

SARS-CoV-2 is an enveloped, positive-sense, single-stranded RNA virus that employs a complicated pattern of replication as well as transcription of genome length and smaller sgRNAs [11]. These sgRNAs are transcriptional intermediates, susceptible to enzymatic degradation, and are not believed to be packaged in the final progeny virion, making them an attractive marker for an actively transcribing virus [12]. Small clinical studies have suggested that, compared with gRNA, sgRNA correlates better with culturable virus. These studies targeted N-gene to detect gRNA and compared sgRNA stability with a relatively less abundant/sensitive E-gene sgRNA assay [2, 3, 9]. Despite the E-gene sgRNA assay possibly being suboptimal and giving false-negative results, these findings have led to the use of sgRNA assay as an outcome in preclinical investigation of novel therapies [13, 14], and the use of sgRNA assay to terminate medical isolation for individuals with coronavirus disease 2019 (COVID-19) has been suggested [3]. In contrast, a recent study found that sgRNAs were detectable up to 17 days after initial detection and that they may be protected from nuclease degradation by double membrane vesicles [15]. However, because this study only had 12 clinical samples, further evidence about the kinetics of sgRNA vs gRNA in longitudinal samples is needed to determine whether sgRNA abundance better reflects recently transcribing viral infection. Additionally, in order to serve as a marker of replicating virus, sgRNA is expected to show a rapid decline after transcriptional inhibition due to ribonuclease degradation, in contrast to gRNA, which may be protected from degradation by viral capsids and therefore persist more durably [16]. Therefore, we hypothesized that, upon treatment with SARS-CoV-2 RNA-dependent RNA polymerase inhibitors [17–19] in cell lines infected with SARS-CoV-2, we should observe a rapid decline of sgRNA after viral death when compared with gRNA.

To address these gaps, we developed an N-gene sgRNA assay to directly compare its stability with N-gene gRNA. sgRNAs in SARS-CoV-2 share a common leader sequence at the 5’ end, which is absent in the gene amplified from the gRNA [9]. We combined the common leader sequence as the forward primer with the Centers for Disease Control and Prevention (CDC) N1 gene assay’s reverse primer [20] to facilitate comparison of N-gene sgRNA with gRNA copies. We applied this assay to serial samples from individuals participating in 2 randomized clinical trials to characterize decay rates. Additionally, we compared the N-gene sgRNA assay with the previously published E-gene sgRNA [3] assay in a subset of randomly selected samples to compare their efficiencies in detecting SARS-CoV-2 sgRNA. Furthermore, we leveraged the inhibition of viral transcription and replication by an RNA-dependent RNA polymerase inhibitor (remdesivir) [19] to measure and compare the decay kinetics of gRNA and sgRNA following polymerase inhibition in SARS-CoV-2-infected A549^ACE2+^ cells.

## METHODS

### Patient Consent

All participants were >18 years of age and provided written informed consent. The studies were approved by the Stanford Institutional Review Board (IRB; #57686 and #58869).

### Overview and Study Population

This was a substudy of 2 phase 2 randomized, placebo-controlled trials of peginterferon-Lambda-1a (Lambda; NCT04331899) and favipiravir (NCT04346628) for the treatment of COVID-19. Individuals >18 years of age with RT-PCR-confirmed SARS-CoV-2 infection were recruited to participate and were eligible if they could be randomized within 72 hours of a positive SARS-CoV-2 test and were not hospitalized. Additional exclusion criteria were respiratory rate <20 breaths per minute, room air oxygen saturation <94%, pregnancy or breastfeeding, or use of other investigational agents for the treatment of COVID-19. In the Lambda trial, enrolled participants were randomized to a single injection with 180 mcg of Lambda vs placebo injection and followed for 28 days. In the favipiravir trial, individuals were randomized to oral favipiravir tablets (1800 mg on day 1, followed by 800 mg twice daily for 9 days) or matching placebo. The primary outcome for both studies was time to cessation of viral shedding, as measured by qRT-PCR performed on oropharyngeal swab samples (Lambda trial) or nasal swabs (favipiravir trial). In August 2020, we amended both protocols to collect nasal swabs (LH-11-10 Longhorn Hydra Sterile Flocked Swab) to assay for gRNA and sgRNA. For the Lambda trial, it was found earlier that in both patients receiving Lambda and placebo, the median time to cessation of viral shedding was 7 days [21]. A single dose of subcutaneous peginterferon Lambda-1a neither shortened the duration of SARS-CoV-2 viral shedding nor improved symptoms in outpatients [21]. The favipiravir trial is an ongoing study and remains blinded.

### Study Population Characteristics

We recruited 205 COVID-19-positive patients from placebo-controlled trials of interferon Lambda (n = 66) and favipiravir (n = 139) between August 2020 and January 2021. All participants were enrolled in the trials within 72 hours of a positive SARS-CoV-2 RT-qPCR test. The median age of the participants (range) was 40 (18–73) years, and 46.8% (96/205) were female. The majority of participants (197/205; 96.1%) reported 1 or more COVID-19-related symptom several days before enrollment (median [range], 5 [0–21] days). Symptoms with onset >3 weeks before study enrollment were not considered to be associated with COVID-19. The most common baseline symptoms reported by the patients before randomization were cough, diarrhea, body ache, headache, fatigue, and shortness of breath (Table 1).

**Table 1.** Characteristics of the Study Participants Recruited From the Lambda and Favipiravir Trials

	Trial		
	Lambda (n = 66)	Favipiravir (n = 139)	Overall (n = 205)
Age, median (IQR), y	36 (31–48.5)	42 (33–53)	40 (32–52)
Female, No. (%)	28 (47.5)	60 (46.2)	88 (46.6)
Symptomatic at enrollment, No. (%)	66 (100)	131 (94.2)	197 (96.1)
Duration of symptoms before enrollment, median (IQR), d	5 (4–7.75)	5 (3–7)	5 (3–7)
0–7 d, No. (%)	49 (74.2)	110 (79.1)	159 (77.6)
8–14 d, No. (%)	14 (21.2)	20 (14.4)	34 (16.6)
15–21 d, No. (%)	3 (4.5)	1 (0.7)	4 (2.0)

Abbreviation: IQR, interquartile range.

Of the 536 swabs collected from 205 patients, 147 patients provided swabs on all 3 days, 37 provided swabs on 2 days, and 21 patients provided a swab on a single day (Supplementary Table 1). Patients for whom the day 1 swab was not available are those who had already enrolled for the trials and only had samples collected on the second or third visit. Patients for whom samples were not available at later time points are those who did not have a swab collected on those days as intended or who dropped from the study before completion.

### RNA Extraction and Quantitative RT-PCR Assay for SARS-CoV-2 RNA

Nasal swabs were collected and transported in 500 µL of Primestore MTM (Longhorn Vaccines & Diagnostics) RNA-stabilizing media. RNA was extracted using the MagMAX Viral/Pathogen Ultra Nucleic Acid Isolation Kit (Cat # A42356 Applied Biosystems) according to the manufacturer’s instructions and eluted in 50 µL of elution buffer. We performed qRT-PCR using the CDC-qualified primers and probes to amplify a 72–base pair product of the N1 region of the SARS-CoV-2 genome [20]. TaqPath 1-step RT-PCR mastermix (Invitrogen, Darmstadt, Germany) was used in a 20-µL reaction volume, and the samples were analyzed on a StepOne-Plus (Applied Biosystems) instrument using the following program: 10 minutes at 50ºC for reverse transcription, followed by 3 minutes at 95ºC and 40 cycles of 10 seconds at 95ºC, 15 seconds at 56ºC, and 5 seconds at 72ºC. We estimated copies/sample from a standard curve using a pET21b+ plasmid (GenScript) with the N-gene. The cycle threshold (Ct) cutoff for positive samples was <38.

### Quantitative RT-PCR Assay for SARS-CoV-2 sgRNA

As all sgRNAs are known to carry a common leader sequence, to amplify N-gene sgRNA we combined a previously described E-gene sgRNA forward primer for the SARS-CoV-2 leader sequence along with the CDC N1-gene segment reverse primer and probe to amplify the 175–base pair N-gene sgRNA product [3]. We used TaqPath 1-step RT-PCR mastermix with 400-nM concentrations of each of the primers and 200 nM of probe to amplify sgRNA. The N-gene PCR reaction conditions were used for sgRNA amplification. We estimated copies/sample from a standard curve using a pET21b+ plasmid with the N-gene sgRNA sequence. The Ct cutoff for positive samples was <38.

The limits of detection (LoDs) for gRNA and sgRNA assays were compared to ensure that they have similar performance. LoDs for both the assays were 10 copies/reaction. The coefficients of correlation (*R*^2^) obtained for the standard curves of 10-fold serial dilution for gRNA and sgRNA were 0.98 and 0.99, respectively. Both the assays had similar slopes on their standard curves (gRNA slope = –2.98; sgRNA slope = –3.07).

On a subset of 35 samples that were positive for N-gene sgRNA, we performed previously reported [3] E-gene sgRNA assays to compare their sensitivities. Briefly, using leader sequence as the forward primer, we performed 1-step RT-PCR with 400-nM concentrations of each of the primers and 200 nM of probe to amplify a 151–base pair product of E-gene sgRNA. Thermal cycling involved 10 minutes at 50ºC for reverse transcription, followed by 3 minutes at 95ºC and 40 cycles of 10 seconds at 95ºC, 15 seconds at 56ºC, and 5 seconds at 72ºC.

### sgRNA Validation by Sanger Sequencing

For the first 15 positive clinical samples, we confirmed amplification product identity by Sanger sequencing. We performed end point PCR using the same primers, purified it by gel electrophoresis, and performed Sanger sequencing with these primers. The resulting sequences were aligned using the SARS-CoV-2 genome (GenBank: MT568638.1) to compare sequence similarity of the product with the leader sequence and N-gene. Samples were considered positive for sgRNA if the leader sequence identity with the reference genome was >98%.

### sgRNA Kinetics in SARS-CoV-2-Infected A549^ACE2+^ Cells

#### Cell Culture and In Vitro SARS-CoV-2 Infection

The human lung epithelial carcinoma cell line A549, overexpressing angiotensin-converting enzyme 2 (ACE2), and A549^ACE2+^ were provided by Ralf Bartenschlager (Heidelberg University) [22]. A549^ACE2+^ cells were cultured in Dulbecco’s Modified Eagle Medium (DMEM; Life Technologies; 11885-092) supplemented with 10% fetal bovine serum (Corning; MT35016CV), 1% penicillin-streptomycin (Thermo Fisher Scientific; 15070063), and 623 µg/mL of Geneticin (Thermo Fisher Scientific; 10121035). For viral infection, cells were seeded a day before infection by culturing 1×10^5^ cells per well in a 6-well plate (Corning). Cells were at passage 14 at the time of infection. Viral infection was performed with the Washington strain of SARS-CoV-2 (2019-nCOV/USA-WA1/2020), titered by plaque assay on VeroE6 cells, at a multiplicity of infection (MOI) of 1. Briefly, in Biosafety level 3 (BSL3) containment, culture media was removed, and cells were washed with phosphate-buffered saline (PBS; Thermo Fisher Scientific; 10-296-028) multiple times before adding the viral stock. Cells were then incubated at 37ºC with 5% CO_2_ for 1 hour while gently rocking. After 1 hour, cells were washed with 1× PBS and incubated in culture media. Supernatant and cells were collected at 1 and 24 hours postinfection (hpi) in TRIzol LS (Thermo Fisher Scientific; 10010023) for RNA extraction. Other wells were either treated with 0.1% final concentration of dimethylsulfoxide (DMSO; Sigma Life Science: Cat# D2650) or 10 µM of remdesivir (Gilead, Cat# NDC 61958-2901-2) in 0.1% DMSO and cultured for longer periods (48, 72, and 96 hpi). It has been previously demonstrated that at a 10-µM prodrug concentration, remdesivir potently inhibits SARS-CoV-2 in A549^ACE2+^ cells [23]. The cytopathic effect on SARS-CoV-2 in vitro–infected A549^ACE2+^ cells that were treated with either 10 µM of remdesivir or vehicle, 0.1% DMSO, was monitored before and after infection. Cell line experiments at all time points and treatment conditions were performed in technical duplicates. Cells and supernatant were collected, and RNA was extracted independently for all technical duplicates without pooling. An image of cells was collected using an EVOS XL core imaging system (Thermo Fisher scientific), with a 10× objective, before collecting cell pellet and supernatant from each treatment and time point.

#### RNA Extraction and RT-PCR

RNA from supernatant and cells collected at 1, 24, 48, 72, and 96 hpi in TRIzol LS was extracted and isolated using standard phenol-chloroform extraction per the manufacturer’s instructions. The SARS-CoV-2 genomic and sgRNA RT-qPCR assays from cell line technical duplicates were further performed in technical duplicates. The RNA copies were quantified using standard curves derived from plasmids. The Eukaryotic 18S rRNA commercial TaqMan assay (4333760T, Thermo Fisher Scientific) was used as an internal control.

### Statistical Analyses

We estimated the change in Ct value for gRNA and sgRNA by day using generalized linear mixed models with a random effect for participant. We tested for differences in the coefficients for collection day for outcomes of gRNA and sgRNA by performing analysis of variance on a joint model with a dummy variable for RNA type. We assessed the correlation between gRNA and sgRNA Ct values using linear mixed models, evaluating marginal and conditional *R*^2^. We used generalized additive mixed models with a random effect for participant to investigate the relationship between sample collection day and Ct values. All analyses were performed using R [24].

## RESULTS

### gRNA and sgRNA RT-qPCR Positivity in Clinical Samples

**Figure 1.**
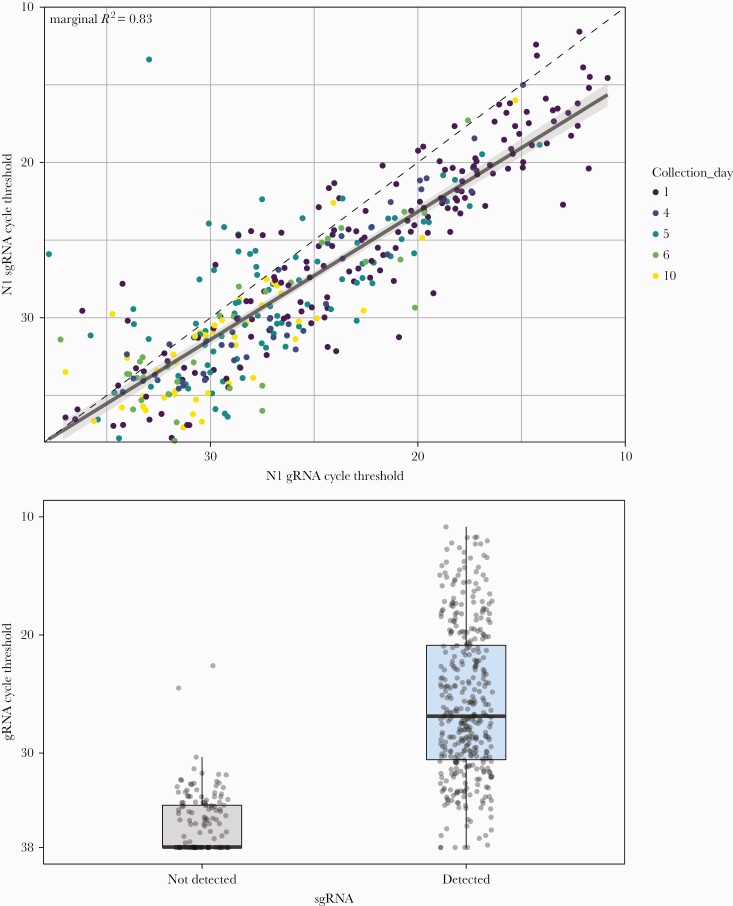
Correlation between genomic and subgenomic RNA in clinical samples. A, Genomic and sgRNA cycle threshold values from clinical samples showed strong correlation (marginal *R*^2^ = 0.83). The dashed line reflects the diagonal of equal cycle thresholds, and the solid line is the best fit regression line, with the shaded area indicating the 95% CI. B, Detection of sgRNA was predicted by cycle threshold value of gRNA. Abbreviations: gRNA, genomic RNA; sgRNA, subgenomic RNA.

We analyzed 536 nasal swab samples collected from 205 COVID-19 patients from the Lambda (n = 177) and favipiravir (n = 359) trials between 0 and 21 days post–symptom onset. For the favipiravir trial, nasal swabs were collected on the day of enrollment (day 1), followed by day 5 and day 10. For the Lambda trial, nasal swabs were collected on the day of enrollment (day 1), followed by day 4 and day 6. Overall gRNA RT-qPCR positivity in samples from the favipiravir trial on days 1, 5, and 10 was 91.5%, 82.9%, and 60.3%, respectively (Table 2). For sgRNA, positivity was 89.2%, 77.2%, and 49.5%. For the Lambda trial, overall gRNA positivity on days 1, 4, and 6 was 91.6%, 90.9%, and 91.9%, respectively. For sgRNA, overall positivity was 81.6%, 74.5%, and 51.6%. We observed a high correlation (marginal *R*^2^ = 0.83) between the cycle threshold (Ct) values of gRNA and sgRNA at all time points, and detection of sgRNA was strongly predicted by gRNA Ct (Figure 1).

**Table 2.** Genomic and sgRNA RT-qPCR Positivity in Longitudinal Samples From the Lambda and Favipiravir Trials

Longitudinal Swab Samples From Lambda Clinical Trial (n = 177)						
	Day 1		Day 4		Day 6	
	Positive, No. (%)	Median Ct	Positive, No. (%)	Median Ct	Positive, No. (%)	Median Ct
gRNA	55/60 (91.6)	24.4	50/55 (90.9%)	30.2	57/62 (91.9%)	33.2
sgRNA	49/60 (81.6)	26.9	41/55 (74.5%)	32.3	32/62(51.6%)	37.3
Longitudinal Swab Samples From Favipiravir Clinical Trial (n = 359)						
	Day 1		Day 5		Day 10	
	Positive, No. (%)	Median Ct	Positive, No. (%)	Median Ct	Positive, No. (%)	Median Ct
gRNA	119/130 (91.5)	23.8	102/123 (82.9)	29.4	64/106 (60.3)	34.4
sgRNA	116/130 (89.2)	25.4	95/123 (77.2)	31.0	51/106 (49.5 )	38

Ct cutoff value for positive samples <38.

Abbreviations: Ct, cycle threshold; gRNA, genomic RNA; RT-qPCR, quantitative reverse transcription polymerase chain reaction; sgRNA, subgenomic RNA.

For the first 15 samples for which sgRNA showed positive amplification, we performed Sanger sequencing. All 15 samples had >98% identity with the SARS-CoV-2 leader sequence, confirming amplification of the sgRNA transcript (Supplementary Figure 1). In a subset of 35 samples in which we performed testing for E-gene sgRNA using a previously published assay [3], we found high correlation (Pearson’s *r* = 0.89) with N-gene sgRNA, but with higher Ct values (median difference, 4.1 cycles) among positive samples. Among 35 samples positive for N-gene sgRNA, 69.0% (24/35) were negative for E-gene sgRNA (Supplementary Figure 2).

Randomization data were available for the Lambda study, while favipiravir still remains blinded.

**Figure 2.**
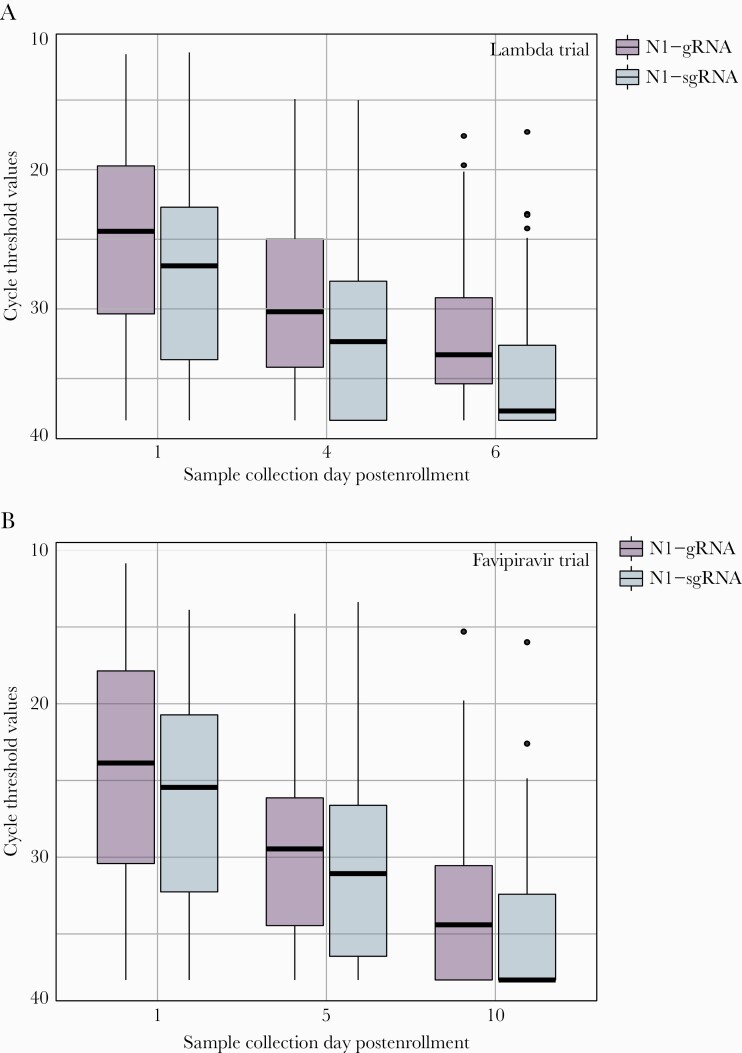
Cycle threshold values for genomic and subgenomic RNA by sample collection day. Boxplot representing cycle threshold values for genomic (purple) and sgRNA (blue) from serially collected COVID-19 samples. A, N-gene genomic and sgRNA decay trend in samples from the Lambda trial collected at days 1, 4, and 6. B, N-gene genomic and sgRNA decay trend in samples from the favipiravir trial collected at days 1, 5, and 10. Abbreviations: COVID-19, coronavirus disease 2019; gRNA, genomic RNA; sgRNA, subgenomic RNA.

**Figure 3.**
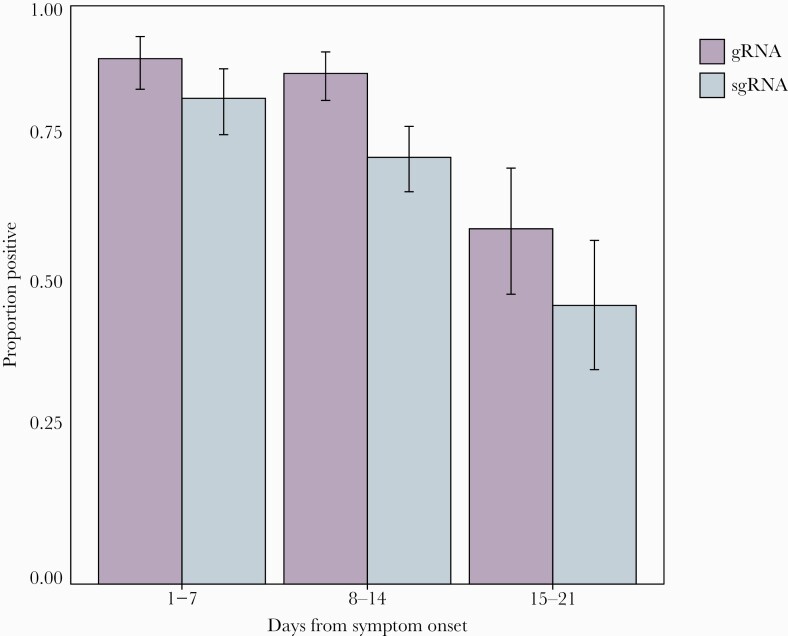
Proportion of samples positive for genomic and subgenomic RNA from time of symptom onset. Samples from the Lambda and favipiravir trials were combined, and a positive sample was one with a cycle threshold <38. Error bars denote 95% exact binomial CIs.

We did not observe any significant difference in the Ct values of sgRNA between Lambda and placebo recipients. At day 6, the gRNA percent positivity was 83.8% (26/31; median Ct value, 32.5) in Lambda and 100% (31/31; median Ct value, 33.4) in the placebo arm. In sgRNA at day 6, percent positivity was 48.3% (15/31; median Ct value, 38.0) in Lambda and 54.8% (17/31; median Ct value, 35.9) in the placebo arm (*P* = .903). In samples in which gRNA was detected but sgRNA was not detected, the median Ct value was 34.4. We found no difference in the rate of Ct value increase by day in gRNA compared with sgRNA in the Lambda trial (1.36 cycles/d vs 1.36 cycles/d; *P* = .97) or favipiravir trial (1.03 cycles/d vs 0.94 cycles/d; *P* = .26) (Figure 2). Among samples collected 15–21 days after symptom onset from both trials combined, sgRNA was detectable in 48.1% (40/83) of participants (Figure 3).

### sgRNA Kinetics in SARS-CoV-2-Infected A549^ACE2+^ Cells Treated With Remdesivir

**Figure 4.**
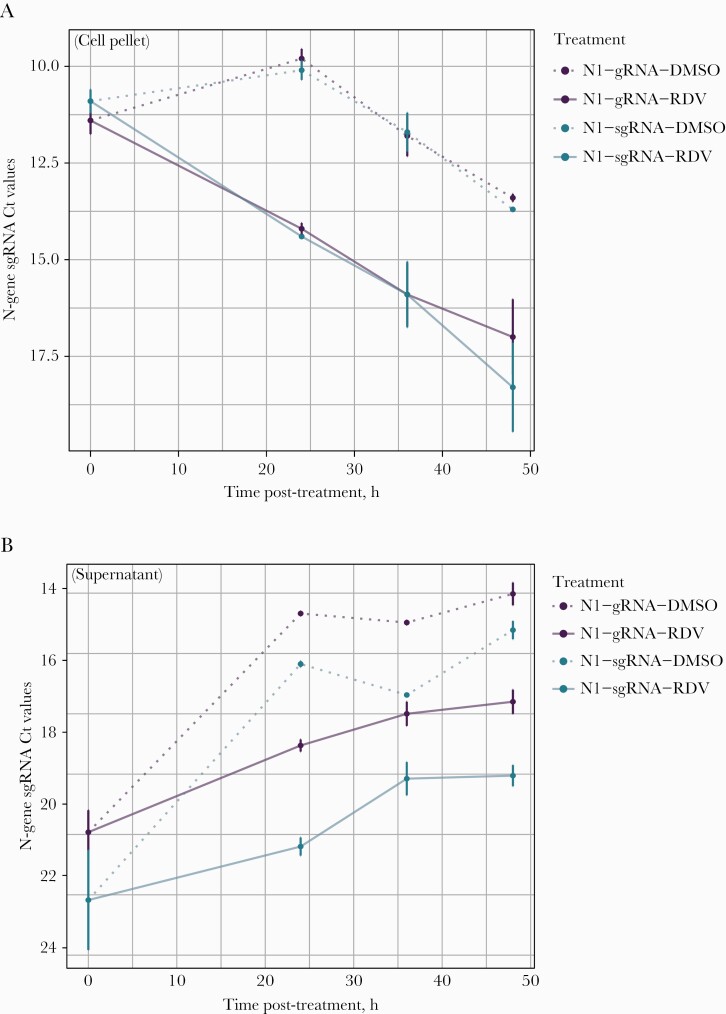
SARS-CoV-2 genomic and subgenomic RNA kinetics in cell culture. SARS-CoV-2 genomic and sgRNA kinetics in A549^ACE2+^ cells treated with 0.1% DMSO and 10 µM of remdesivir. Solid lines indicate genomic (purple) and sgRNA (blue) levels in A549^ACE2+^ cells treated with remdesivir. Dotted lines indicate genomic (purple) and sgRNA (blue) levels in cells treated with 0.1% DMSO. A, Corresponds to genomic and sgRNA degradation kinetics in washed cell pellets from the A549^ACE2+^ cell line. B, Corresponds to genomic and sgRNA degradation kinetics in supernatant from the A549^ACE2+^ cell line. Abbreviations: DMSO, dimethylsulfoxide; gRNA, genomic RNA; RDV, remdesivir; SARS-CoV-2, severe acute respiratory syndrome coronavirus 2; sgRNA, subgenomic RNA.

We compared SARS-CoV-2 gRNA and sgRNA degradation kinetics after transcriptional inhibition by the antiviral drug remdesivir. We treated SARS-CoV-2-infected A549^ACE2+^ cells with 0.1% DMSO vehicle control and 10 µM of remdesivir at 24 hpi. Cytopathic effects were observed in cells treated with DMSO control but not remdesivir (Supplementary Figure 3). Compared with DMSO-treated cells, SARS-CoV-2 replication in remdesivir-treated cells was markedly reduced (nadir gRNA Ct, 9.6 vs 14.2; nadir sgRNA Ct, 10.0 vs 14.5). In remdesivir-treated cells, gRNA and sgRNA Ct values rose at similar rates in cells (0.11/h vs 0.09/h; *P* = .153) and declined by similar rates in supernatant (–0.06/h vs 0.06/h; *P* = .914) (Figure 4).

## DISCUSSION

While there has been considerable interest in the use of sgRNAs as markers of replicating SARS-CoV-2 infection, evidence concerning the decay of sgRNA following onset of infection in humans and cell culture has been lacking. Using longitudinal samples from 2 clinical trials, we found that sgRNA was detectable in 46% of participants from the Lambda trial and 50% from the favipiravir trial 15–21 days after symptom onset. While gRNA was detectable for longer than sgRNA, they were highly correlated and had indistinguishable rates of decline within individuals over time. We found consistent results in cell culture, whereby gRNA and sgRNA copies declined at the same rate following inhibition of transcription by remdesivir. Taken together, these findings suggest that detection of sgRNAs is not a reliable marker of recent viral transcription and does not provide marginal information over quantification of gRNA. Earlier findings of greater specificity of sgRNAs than gRNAs compared with a reference standard of culture may be explained by the lower analytical sensitivity of the sgRNA assays [3, 9, 10], particularly using less sensitive E-gene assays.

RNA transcripts have been used as markers of viability or metabolic activity for a number of bacterial and viral pathogens. In bacteria, mRNAs have much shorter half-lives than DNA due to degradation by ribonucleases, such that their presence indicates recent metabolic activity [25–27]. Similarly, RNA transcription assays have been used to assess replication-competent viral pool size for HIV-1 [28]. For SARS-CoV-2, sgRNA transcription is believed to occur inside double-membrane vesicles, which may protect viral genomic and subgenomic RNA from cytoplasmic degradation due to host enzymes [29–33]. We found that sgRNA and gRNA increased at the same rate following infection of cells and then declined at the same rate following cell death (in the control cells treated with DMSO) or following inhibition of RDRP (RNA-dependent RNA polymerase) by remdesivir. If sgRNAs were rapidly degraded by ribonucleases, we would have expected a more rapid decline in sgRNAs compared with gRNAs, but this was not observed. Similarly, in the supernatant, we saw no difference in change in sgRNAs compared with gRNAs, again failing to identify rapid clearance of sgRNAs by ribonucleases. Several previous studies have targeted E-gene sgRNA, reporting in small clinical series that these correlated well with culture [34, 35]. This finding may be explained by the fact that E-gene transcripts are less abundant than N-gene transcripts, so assays targeting them will have lower analytical sensitivity [36]. Indeed, we performed direct comparison of N-gene and E-gene assays and found that the latter were ~5 Ct values higher for the same sample. There is not a clear premise to infer that E-gene transcripts are more rapidly degraded by ribonucleases or that they better reflect recent transcription than N-gene sgRNAs. Negative-strand RNA detection in SARS-CoV-2 has recently been reported as another potential viability marker [37]. In a study by Alexanderson et al., negative strand RNA was detected up to 11 days [15]. However, negative strand assays may be less analytically sensitive than sgRNA assays [37]. This could make them appear to be more specific, compared with culture, in clinical samples as they are more likely to be negative when viral abundance is low. While we found gRNA positivity to be higher than sgRNA positivity at later time points, we believe this is due to the slightly higher abundance of gRNA, as gRNA detection in these discrepant samples was just above the limit of detection (median Ct value, 34.4).

Our study has several limitations, which include lack of viral culture data and samples at later time points. Additionally, to study the sgRNA decay kinetics, we targeted a single and highly abundant gene to compare and quantify gRNA and sgRNA, though we found high correlation between E-gene and N-gene sgRNA copy numbers. Analyzing additional targets would help us understand the stability of other sgRNAs. Another limitation of our study is the lack of unblinded data from the favipiravir study, precluding analysis by study arm. Favipiravir is substrate for the RNA-dependent RNA-polymerase (RDRP) enzyme, which is mistaken by the enzyme as a purine nucleotide, thus inhibiting its activity, leading to termination of viral protein synthesis [38]. Because inactivation of RDRP prevents viral transcription and replication, production of both gRNAs and sgRNAs is inhibited, such that differences in sgRNA and sgRNA levels could be driven be differential decay. To evaluate this possibility, we performed in vitro experiments with another RDRP inhibitor, remdesivir. While remdesivir inhibited viral replication and sgRNA production as expected, we found no differences in gRNA and sgRNA decay rates. It is possible that favipiravir led to differential sgRNA and gRNA decline in vivo, despite there having been no differences in the combined clinical cohort (which would require an effect in the antiviral arm and no effect in the placebo arm). We therefore cannot draw conclusions about the effects of this antiviral drug in vivo. Nevertheless, we believe that the lack of effects in the combined group in the favipiravir trial and each arm of the Lambda trial suggest that during natural infection, in the absence of antivirals, gRNA and sgRNA levels do not decline at differential rates. Moreover, the prolonged detection of sgRNA (>50% of samples collected >14 days after symptom onset had detectable sgRNA), despite epidemiologic studies showing little transmission after 10 days, does not support sgRNA as a marker of infectiousness.

In summary, we found that SARS-CoV-2 sgRNAs are persistently detectable in clinical samples, correlate strongly with gRNA, and decline at indistinguishable rates in clinical samples and cell culture. We find little evidence to support the premise that sgRNA detection is a reliable marker of transcriptionally active virus or that it provides additional information beyond detection of gRNA in clinical samples. We advise caution against using sgRNA assays to inform decisions concerning treatment or medical isolation.

## Supplementary Data

Supplementary materials are available at *Open Forum Infectious Diseases* online. Consisting of data provided by the authors to benefit the reader, the posted materials are not copyedited and are the sole responsibility of the authors, so questions or comments should be addressed to the corresponding author.

ofab310_suppl_Supplementary_Figure_S1

ofab310_suppl_Supplementary_Figure_S2

ofab310_suppl_Supplementary_Figure_S3

ofab310_suppl_Supplementary_Materials

## References

[1] He X , Lau EHY, Wu P, et al. Temporal dynamics in viral shedding and transmissibility of COVID-19. Nat Med 2020; 26:672–5. 32296168 10.1038/s41591-020-0869-5

[2] Kim MC , Cui C, Shin KR, et al. Duration of culturable SARS-CoV-2 in hospitalized patients with Covid-19. N Engl J Med 2021; 384:671–3. 33503337 10.1056/NEJMc2027040 PMC7934323

[3] Wölfel R , Corman VM, Guggemos W, et al. Virological assessment of hospitalized patients with COVID-2019. Nature 2020; 581:465–9. 32235945 10.1038/s41586-020-2196-x

[4] Mallett S , Allen AJ, Graziadio S, et al. At what times during infection is SARS-CoV-2 detectable and no longer detectable using RT-PCR-based tests? A systematic review of individual participant data. BMC Med 2020; 18:346. 33143712 10.1186/s12916-020-01810-8 PMC7609379

[5] Bullard J , Dust K, Funk D, Strong JE, et al. Predicting infectious SARS-CoV-2 from diagnostic samples. Clin Infect Dis. In press. 10.1093/cid/ciaa638 PMC7314198 32442256

[6] COVID-19 Investigation Team. Clinical and virologic characteristics of the first 12 patients with coronavirus disease 2019 (COVID-19) in the United States. Nat Med 2020; 26:861–8. 32327757 10.1038/s41591-020-0877-5 PMC12755114

[7] Singanayagam A , Patel M, Charlett A, et al. Duration of infectiousness and correlation with RT-PCR cycle threshold values in cases of COVID-19, England, January to May 2020. Euro Surveill 2020; 25:2001483. 10.2807/1560-7917.ES.2020.25.32.2001483 PMC7427302 32794447

[8] Jafari H , Amiri Gharaghani M. Cultural challenges: the most important challenge of COVID-19 control policies in Iran. Prehosp Disaster Med 2020; 35:470–1. 32434624 10.1017/S1049023X20000710 PMC7264455

[9] Perera RAPM , Tso E, Tsang OTY, et al. SARS-CoV-2 virus culture and subgenomic RNA for respiratory specimens from patients with mild coronavirus disease. Emerg Infect Dis 2020; 26:2701–4. 32749957 10.3201/eid2611.203219 PMC7588524

[10] Williamson BN , Feldmann F, Schwarz B, et al. Clinical benefit of remdesivir in rhesus macaques infected with SARS-CoV-2. Nature 2020; 585:273–6. 32516797 10.1038/s41586-020-2423-5 PMC7486271

[11] Sola I , Almazán F, Zúñiga S, Enjuanes L. Continuous and discontinuous RNA synthesis in coronaviruses. Annu Rev Virol 2015; 2:265–88. 26958916 10.1146/annurev-virology-100114-055218 PMC6025776

[12] Wu HY , Brian DA. Subgenomic messenger RNA amplification in coronaviruses. Proc Natl Acad Sci U S A 2010; 107:12257–62. 20562343 10.1073/pnas.1000378107 PMC2901459

[13] Corbett KS , Flynn B, Foulds KE, et al. Evaluation of the mRNA-1273 vaccine against SARS-CoV-2 in nonhuman primates. N Engl J Med 2020; 383:1544–55. 32722908 10.1056/NEJMoa2024671 PMC7449230

[14] van Doremalen N , Lambe T, Spencer A, et al. ChAdOx1 nCoV-19 vaccine prevents SARS-CoV-2 pneumonia in rhesus macaques. Nature 2020; 586:578–82. 32731258 10.1038/s41586-020-2608-y PMC8436420

[15] Alexandersen S , Chamings A, Bhatta TR. SARS-CoV-2 genomic and subgenomic RNAs in diagnostic samples are not an indicator of active replication. Nat Commun 2020; 11:6059. 33247099 10.1038/s41467-020-19883-7 PMC7695715

[16] Cliver DO . Capsid and infectivity in virus detection. Food Environ Virol 2009; 1:123–8. 20234879 10.1007/s12560-009-9020-y PMC2837222

[17] Yin W , Luan X, Li Z, et al. Structural basis for inhibition of the SARS-CoV-2 RNA polymerase by suramin. Nat Struct Mol Biol 2021; 28:319–25. 33674802 10.1038/s41594-021-00570-0

[18] Naydenova K , Muir KW, Wu LF, et al. Structure of the SARS-CoV-2 RNA-dependent RNA polymerase in the presence of favipiravir-RTP. Proc Natl Acad Sci U S A 2021; 118. 10.1073/pnas.2021946118 PMC7896311 33526596

[19] Kokic G , Hillen HS, Tegunov D, et al. Mechanism of SARS-CoV-2 polymerase stalling by remdesivir. Nat Commun 2021; 12:279. 33436624 10.1038/s41467-020-20542-0 PMC7804290

[20] Centers for Disease control and Prevention. Research Use Only 2019-Novel Coronavirus (2019-nCoV) Real-time RT-PCR Primers and Probes. Updated June 6, **2020**. https://www.cdc.gov/coronavirus/2019-ncov/lab/rt-pcr-panel-primer-probes.html.

[21] Jagannathan P , Andrews JR, Bonilla H, et al. Peginterferon Lambda-1a for treatment of outpatients with uncomplicated COVID-19: a randomized placebo-controlled trial. Nat Commun 2021; 12:1967. 33785743 10.1038/s41467-021-22177-1 PMC8009873

[22] Klein S , Cortese M, Winter SL, et al. SARS-CoV-2 structure and replication characterized by in situ cryo-electron tomography. Nat Commun 2020; 11:5885. 33208793 10.1038/s41467-020-19619-7 PMC7676268

[23] de Vries M , Mohamed AS, Prescott RA, et al. A comparative analysis of SARS-CoV-2 antivirals characterizes 3CLpro inhibitor PF-00835231 as a potential new treatment for COVID-19. J Virol. In press. 10.1128/JVI.01819-20 PMC8139662 33622961

[24] R Core Team. R: A language and environment for statistical computing. Vienna, Austria: R Foundation for Statistical Computing, 2018. Available at: https://www.R-project.org/.

[25] Cenciarini-Borde C , Courtois S, La Scola B. Nucleic acids as viability markers for bacteria detection using molecular tools. Future Microbiol 2009; 4:45–64. 19207099 10.2217/17460913.4.1.45

[26] Nocker A , Camper AK. Novel approaches toward preferential detection of viable cells using nucleic acid amplification techniques. FEMS Microbiol Lett 2009; 291:137–42. 19054073 10.1111/j.1574-6968.2008.01429.x

[27] Neidhardt FC , Magasanik B. Studies on the role of ribonucleic acid in the growth of bacteria. Biochim Biophys Acta 1960; 42:99–116. 13728193 10.1016/0006-3002(60)90757-5

[28] Plantin J , Massanella M, Chomont N. Inducible HIV RNA transcription assays to measure HIV persistence: pros and cons of a compromise. Retrovirology 2018; 15:9. 29343255 10.1186/s12977-017-0385-y PMC5773137

[29] Escors D , Izeta A, Capiscol C, Enjuanes L. Transmissible gastroenteritis coronavirus packaging signal is located at the 5’ end of the virus genome. J Virol 2003; 77:7890–902. 12829829 10.1128/JVI.77.14.7890-7902.2003 PMC161917

[30] Snijder EJ , Limpens RWAL, de Wilde AH, et al. A unifying structural and functional model of the coronavirus replication organelle: tracking down RNA synthesis. PLoS Biol 2020; 18:e3000715. 32511245 10.1371/journal.pbio.3000715 PMC7302735

[31] Wolff G , Limpens RWAL, Zevenhoven-Dobbe JC, et al. A molecular pore spans the double membrane of the coronavirus replication organelle. Science 2020; 369:1395–8. 32763915 10.1126/science.abd3629 PMC7665310

[32] Wada M , Lokugamage KG, Nakagawa K, et al. Interplay between coronavirus, a cytoplasmic RNA virus, and nonsense-mediated mRNA decay pathway. Proc Natl Acad Sci U S A 2018; 115:E10157–66. 30297408 10.1073/pnas.1811675115 PMC6205489

[33] Wu HY , Brian DA. Subgenomic messenger RNA amplification in coronaviruses. Proc Natl Acad Sci U S A 2010; 107:12257–62. 20562343 10.1073/pnas.1000378107 PMC2901459

[34] Perera RAPM , Tso E, Tsang OTY, et al. SARS-CoV-2 virus culture and subgenomic RNA for respiratory specimens from patients with mild coronavirus disease. Emerg Infect Dis 2020; 26:2701–4. 32749957 10.3201/eid2611.203219 PMC7588524

[35] Williamson BN , Feldmann F, Schwarz B, et al. Clinical benefit of remdesivir in rhesus macaques infected with SARS-CoV-2. Nature 2020; 585:273–6. 32516797 10.1038/s41586-020-2423-5 PMC7486271

[36] Kim D , Lee JY, Yang JS, et al. The architecture of SARS-CoV-2 transcriptome. Cell 2020; 181:914–21.e10. 32330414 10.1016/j.cell.2020.04.011 PMC7179501

[37] Hogan CA , Huang C, Sahoo MK, et al. Strand-specific reverse transcription PCR for detection of replicating SARS-CoV-2. Emerg Infect Dis 2021; 27:632–5. 33496233 10.3201/eid2702.204168 PMC7853532

[38] Agrawal U , Raju R, Udwadia ZF. Favipiravir: a new and emerging antiviral option in COVID-19. Med J Armed Forces India 2020; 76:370–6. 32895599 10.1016/j.mjafi.2020.08.004 PMC7467067

