# Supplementary figures and images for: SARS-CoV-2 Subgenomic RNA Kinetics in Longitudinal Clinical Samples
Source: Open Forum Infect Dis. 2021 Jun 11;8(7):ofab310. doi: 10.1093/ofid/ofab310 (PMC8291522; doi:10.1093/ofid/ofab310)

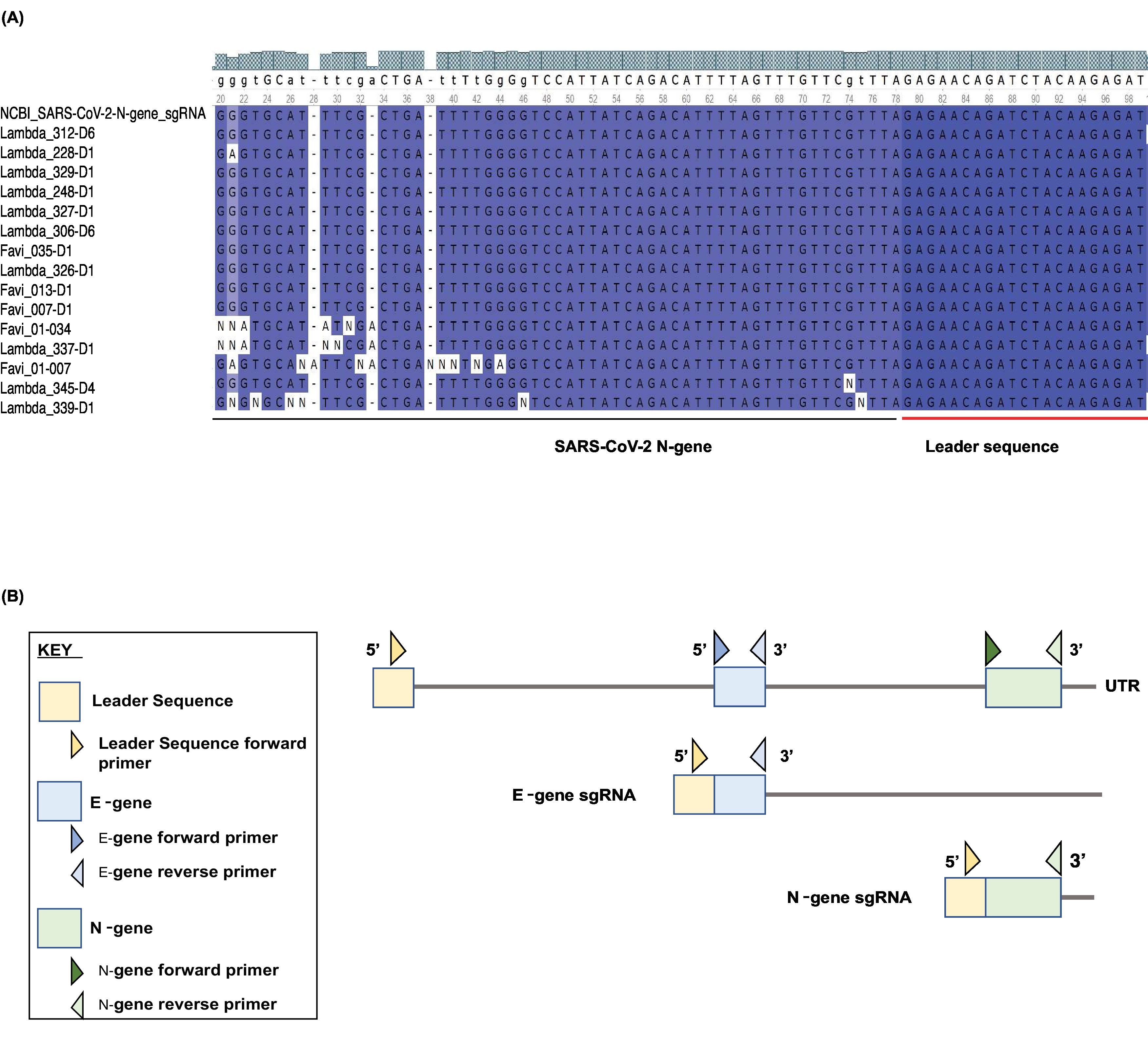

Supplement: ofab310_suppl_Supplementary_Figure_S1 [file ofab310_suppl_supplementary_figure_s1.jpeg]

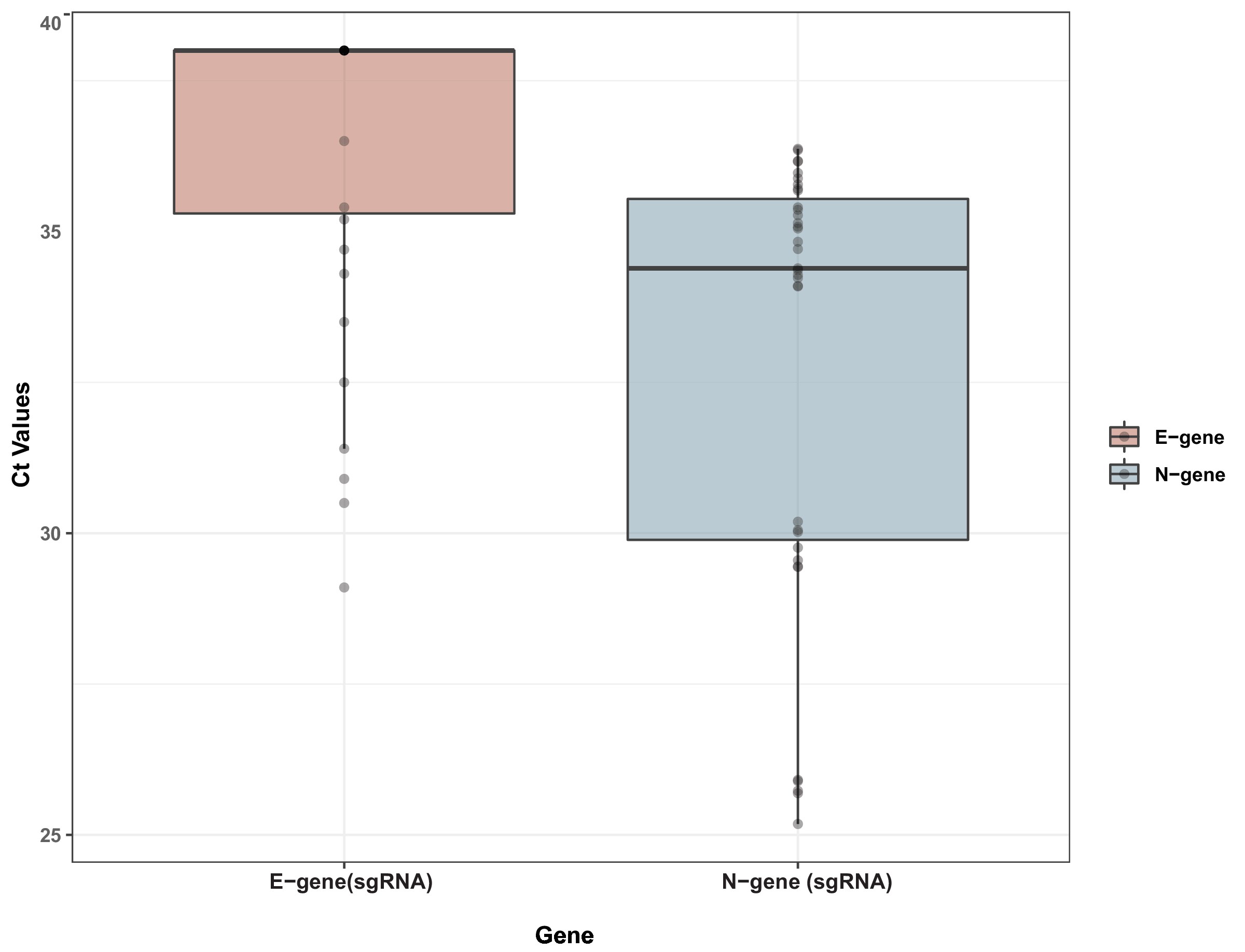

Supplement: ofab310_suppl_Supplementary_Figure_S2 [file ofab310_suppl_supplementary_figure_s2.jpeg]

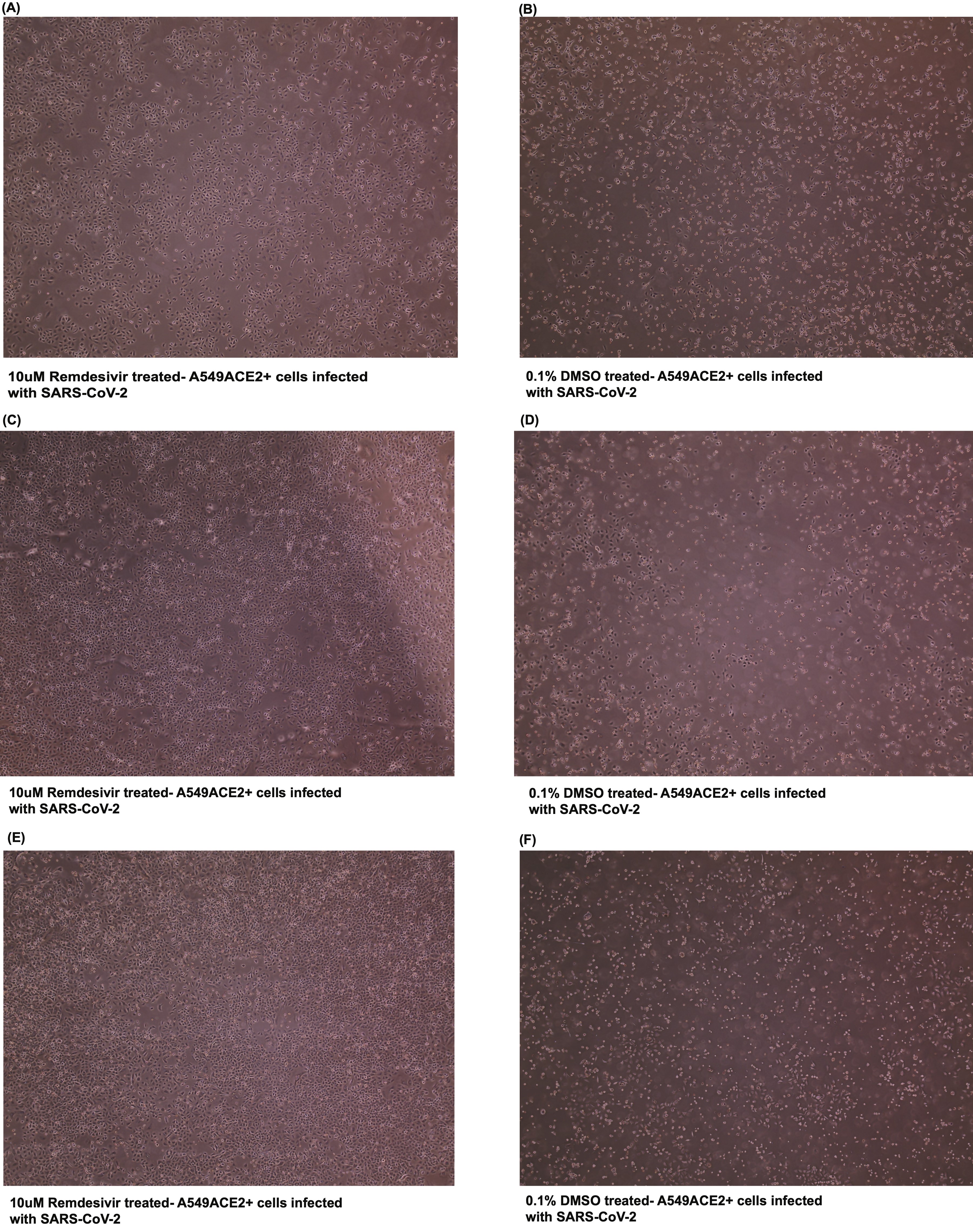

Supplement: ofab310_suppl_Supplementary_Figure_S3 [file ofab310_suppl_supplementary_figure_s3.jpeg]
