# Supplementary material for: SARS-CoV-2 Subgenomic RNA Kinetics in Longitudinal Clinical Samples
Source: Open Forum Infect Dis. 2021 Jun 11;8(7):ofab310. doi: 10.1093/ofid/ofab310 (PMC8291522; doi:10.1093/ofid/ofab310)
Supplement: ofab310_suppl_Supplementary_Materials [file ofab310_suppl_supplementary_materials.pdf]

Supplementary Table 2: Distribution of nasal swab samples from favipiravir and Lambda trials based on sample availability.

	Nasal swab availability		
	**All three days**	**Two days**	**Single day**
**favipiravir**	294 (98 patients)	48 (24 patients)	17 (17 patients)
**Day 1**	98	21	11
**Day 5**	98	19	6
**Day 10**	98	8	0
**Lambda**	147 (49 patients)	26 (13 patients)	4 (4 patients)
**Day 1**	49	9	2
**Day 4**	49	6	0
**Day 6**	49	11	2
